# Mathematical evaluation of community level impact of combining bed nets and indoor residual spraying upon malaria transmission in areas where the main vectors are *Anopheles arabiensis* mosquitoes

**DOI:** 10.1186/1756-3305-6-17

**Published:** 2013-01-16

**Authors:** Fredros O Okumu, Samson S Kiware, Sarah J Moore, Gerry F Killeen

**Affiliations:** 1 Environmental Health and Ecological Sciences Thematic Group, Ifakara Health Institute, Ifakara, Tanzania; 2 Department of Diseases Control, London School of Hygiene and Tropical Medicine, London, UK; 3 Department of Mathematics, Statistics, and Computer Science, Marquette University, Milwaukee, USA; 4 Vector Biology Department, Liverpool School of Tropical Medicine, Liverpool, UK

## Abstract

**Background:**

Indoor residual insecticide spraying (IRS) and long-lasting insecticide treated nets (LLINs) are commonly used together even though evidence that such combinations confer greater protection against malaria than either method alone is inconsistent.

**Methods:**

A deterministic model of mosquito life cycle processes was adapted to allow parameterization with results from experimental hut trials of various combinations of untreated nets or LLINs (Olyset®, PermaNet 2.0®, Icon Life® nets) with IRS (pirimiphos methyl, lambda cyhalothrin, DDT), in a setting where vector populations are dominated by *Anopheles arabiensis*, so that community level impact upon malaria transmission at high coverage could be predicted.

**Results:**

Intact untreated nets alone provide equivalent personal protection to all three LLINs. Relative to IRS plus untreated nets, community level protection is slightly higher when Olyset® or PermaNet 2.0® nets are added onto IRS with pirimiphos methyl or lambda cyhalothrin but not DDT, and when Icon Life® nets supplement any of the IRS insecticides. Adding IRS onto any net modestly enhances communal protection when pirimiphos methyl is sprayed, while spraying lambda cyhalothrin enhances protection for untreated nets but not LLINs. Addition of DDT reduces communal protection when added to LLINs.

**Conclusions:**

Where transmission is mediated primarily by *An. arabiensis,* adding IRS to high LLIN coverage provides only modest incremental benefit (e.g. when an organophosphate like pirimiphos methyl is used), but can be redundant (e.g. when a pyrethroid like lambda cyhalothin is used) or even regressive (e.g. when DDT is used for the IRS). Relative to IRS plus untreated nets, supplementing IRS with LLINs will only modestly improve community protection. Beyond the physical protection that intact nets provide, additional protection against transmission by *An. arabiensis* conferred by insecticides will be remarkably small, regardless of whether they are delivered as LLINs or IRS. The insecticidal action of LLINs and IRS probably already approaches their absolute limit of potential impact upon this persistent vector so personal protection of nets should be enhanced by improving the physical integrity and durability. Combining LLINs and non-pyrethroid IRS in residual transmission systems may nevertheless be justified as a means to manage insecticide resistance and prevent potential rebound of not only *An. arabiensis*, but also more potent, vulnerable and historically important species such as *Anopheles gambiae* and *Anopheles funestus*.

## Background

Long-lasting insecticide treated nets (LLINs) and indoor residual spraying (IRS) with persistent insecticides are by far the most common malaria prevention methods, and have resulted in significant decline of morbidity and mortality in many countries [1,2]. The two methods are often used together in the same households with the aim of achieving greater impact than either method alone, especially in highly endemic areas or in epidemic situations. However, given the massive financial and logistical requirements of implementing either strategy, much less both, LLIN-IRS combinations are increasingly scrutinized to determine whether there are any additional benefits relative to using just LLINs or IRS alone and whether these marginal benefits would be lower or higher than the costs involved. A major challenge in this regard has been the shortage of empirical evidence to ascertain potential synergies or redundancies in combining these two methods [3].

In response, a number of trials are now being reported that address this question but the evidence until now has been mixed. While the only randomized controlled trial conducted so far [4] has shown no additional benefits of the combinations relative to individual interventions, observational studies of non-randomized programmatic applications [5], and at least one experimental hut study [6] have reported apparent improvements when LLINs and IRS are combined. Besides, an earlier review of many previous malaria control programs showed that while LLIN-IRS combinations appear advantageous in some scenarios, this was not a consistent outcome as there were many other situations without such benefits [3,7].

In a recent experimental hut evaluation of multiple combinations of LLIN types and IRS insecticides in a rural Tanzanian village, where *Anopheles arabiensis* was the main malaria vector, it was observed that adding IRS into houses with current LLINs does not enhance personal protection and only modestly increases mosquito mortality even where highly mosquitocidal non-pyrethroids such as the organophosphate, pirimiphos-methyl are used for IRS (Okumu *et al.,* unpublished). That study also showed that adding intact bed nets onto IRS enhances personal protection by preventing mosquito bites, even if the nets are non-insecticidal (i.e. untreated), and by slightly increasing vector mortality if LLINs are used.

The number of field trials conducted on scales large enough to capture the full community-level impacts of LLINs, IRS or combinations thereof, are and will remain limited due to cost and practicality. Mathematical models are therefore increasingly viewed as being useful for extrapolating results from household-level experimental hut trials (which can be done affordably and in high-throughput), to simulate expected community-levels effects of such intervention options [3,8-11]. In this article, we adapt an existing deterministic model of the life-cycle processes of mosquitoes and sporogonic-stage malaria parasites, to enable ready parameterization directly from experimental hut data, so that the likely community-wide impacts of using LLINs or untreated nets together with IRS can be assessed. The model is then applied to predict the likely community-level impact of specific combinations of nets and IRS based on locally executed experimental hut trials (Okumu *et al.,* unpublished), in a rural Tanzanian village where malaria transmission is dominated by *Anopheles arabiensis* following the near-elimination of *Anopheles gambiae sensu stricto* by high LLIN usage rates in the area [12,13].

## Methods

### Model description

A detailed description of this static, deterministic model, which has been incrementally improved over time, as well as details of its earlier applications, can be obtained from previous publications [11,14,15]. The model version applied here is an improvement of versions that have previously been used for a number of purposes including *inter alia*: 1) to compare impacts of LLINs when targeted to all age-groups as opposed to coverage of only pregnant women and children [16], 2) to estimate effects of combining LLINs with odour-baited mosquito traps [15], 3) to assess the extent of exposure to malaria that occurs outside human houses [17], and 4) to assess tradeoffs between repellent and toxic properties of vector control insecticides [11].

Modifications to the most recent formulation [11,14] were introduced to enable direct input of data from standard experimental hut evaluations of intradomicilliary vector control methods [18,19]. Unlike all previous versions, these latest modifications recognize the fact that untreated mosquito nets, commonly used as ‘experimental controls’ in hut studies actually also provide substantial basic protection simply by physically obstructing mosquitoes attempting to bite people sleeping under the nets.

**Figure 1:**
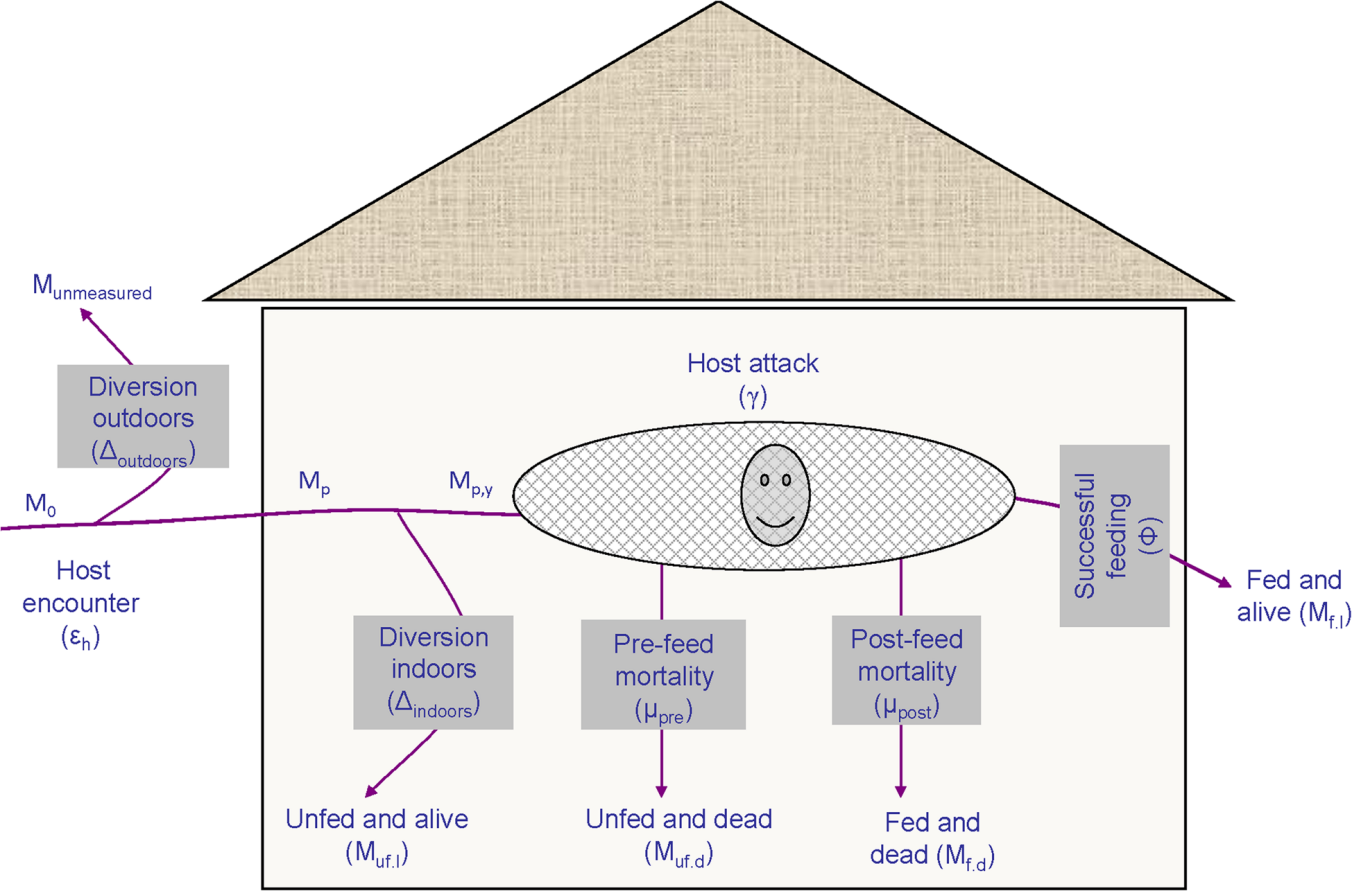
**Diagrammatic representation of mosquito host seeking processes as quantifiable in standard experimental hut studies.** All mosquitoes caught in the huts and in exit traps attached to the hut are considered as having entered the hut and therefore not diverted outdoors. *M*_*p*_ refers to mosquitoes entering the huts while M_p,y_ refers to mosquitoes attacking the host (in this case, human hosts) inside the hut. Where there is no intervention at all or where only untreated nets are used, the total number of mosquitoes entering the huts would be expected to be *M*_*0*_ or *M*_*1*_ respectively.

To represent total protection attainable from IRS or nets, the process leading to attack and feeding by host-seeking mosquitoes was redefined such that for vector control interventions that can divert mosquitoes from actually reaching a human host inside a house, the diversion process was subdivided into two phases (Figure 1). The first is the diversion that occurs outdoors as mosquitoes attempt to enter the house with the intervention (Δ_outdoors_), and the second is the diversion that occurs indoors when the mosquito has already entered the house to attack a human inside (Δ_indoors_). For practical purposes, the diversion outdoors may be considered as a long-range diversion since it occurs at greater distances than the diversion indoors, which may be considered short-range. Therefore, using the example of a bed net as a personal protection measure, we can say that for a mosquito to successfully attack any human using a bed net, i.e. an attack upon a protected net user, that mosquito must not have been diverted at long range outdoors prior to entering the house, and it must also not have been diverted at short range indoors prior to biting the net user. These two distinct and measurable [3,18,20] proportional diversion parameters therefore represent underlying determinants of malaria transmission at individual and community level, which typically have quite low values that can be readily increased by protective interventions such as nets or sprays [11]. While it is acknowledged that a variable minority of female malaria vectors appear to enter huts only to rest in a given night [21], it is assumed that this fraction of the sampled mosquito population is negligible. Based on recent observations in rural Tanzania, where most of the mosquitoes caught in experimental huts were unfed (Okumu *et al.,* unpublished), it is further assumed that the vast majority of vectors entering human-occupied houses do so with a sole intention of attacking and obtaining blood meals from the human hosts inside.

Unlike in previous models wherein the second level of diversion (Δ_indoors_) was not explicitly identified [15,22], the attack probability is hereby redefined as the remaining fraction of mosquitoes encountering the host-occupied house that are unaffected by either of these two sequential diversionary processes:

(1) $$\gamma =\left(1-\text{Δoutdoors}\right)\left(1-\text{Δindoors}\right)$$

The revision allows us to unambiguously distinguish long-range spatial repellence, where mosquitoes are diverted at a distance before they enter huts, from short range deterrence and contact irritant effects, where the interventions force mosquitoes that come into huts to exit those huts without feeding [3,23]. Due to ethical constraints upon using a truly representative negative control with no protection whatsoever [24], the field experimentations (Okumu *et al.,* unpublished), from which we draw the data for the simulations reported here, were conducted using experimental huts [20] in which human volunteers were provided with intact untreated nets as controls, instead of absolute ‘zero-protection’ controls. For purposes of this model, it is therefore assumed that in houses where the only intervention used is untreated nets, i.e. where there is no chemical-induced long-range repellence or physical barriers such as screens on eaves and windows, no diversion occurs outdoors (Δ_outdoors_ = 0) and therefore the number of mosquitoes caught inside those huts would be approximately similar to the number caught in houses with no intervention at all.

The total catch of a given malaria vector species in a given experimental hut or set of huts having a given protective indoor intervention (*M*_*p*_) can therefore be specified for two important baseline protection scenarios (*p*) with either no protection at all (*p* = 0), or protection with an untreated net alone (*p* = 1):

(2) $$M_{0}\approx M_{1}>1$$

where *M*_*0*_ specifies the total catch of the same vector species that would be obtained in the same experimental hut or huts if no protective intervention was used (true negative control, *p* = 0) and *M*_*1*_ refers to the total catch of the same vector species that would be obtained in the same experimental hut or huts if only an untreated bed net was used (pseudo-negative control, *p = 1*). Note that because these mosquito count parameters *(M)* refer only to counts of mosquitoes within or exiting from experimental huts, this use of the multi-level subscript, *p,* to denote alternative protection option scenarios is consistent with previous definitions, which specify single protection option against indoor bites only [11]. The interpretation of equations 1 and 2, is however, subject to one obvious constraint, that is, where the long range diversion outdoors is negative, its value should be specified as zero, otherwise, the measured value is specified.

The diversion that occurs prior to house entry, for any other protective measure, is therefore approximated by using mosquito catch data from houses with untreated nets rather than the absolute negative control.

(3) $$\text{Δoutdoors}=1-\frac{\text{Mp}}{M0}\approx 1-\frac{\text{Mp}}{M1}$$

Once mosquitoes have entered an experimental hut, it is assumed that all will be successfully collected and classified based on their physiological and vital status, as either: 1) unfed and alive *(M*_*uf.l*_*)*, meaning that they did not attack the host inside the hut and are therefore assumed to have been deterred from attacking, 2) unfed and dead *(M*_*uf.d*_*)*, meaning they attacked the host and died in the process without obtaining a blood meal, 3) fed and alive *(M*_*f.l*_*)*, meaning that they attacked the host but survived and successfully obtained a blood meal, or 4) fed and dead *(M*_*f.d*_*)*, also meaning that they attacked the host, successfully obtained a blood meal but then died, presumably as a result of the attack (Figure 1):

(4) Mp=Muf,l+Muf,d+Mf,l+Mf,d

The diversion that occurs indoors is calculated to represent the number of malaria vector mosquitoes that enter the huts but do not attack the host:

(5) $$\text{Δindoors}=1-\frac{\text{Mp},\gamma }{\text{Mp}}$$

where *M*_*p,γ*_ refers to the total number of malaria vectors that are considered to have entered the huts and attacked the human inside that hut, M_P_ ≥ 0, and M_p,y_ ≤ M_p._ The parameter, *M*_*p,γ*_ therefore includes both fatal attacks, represented by dead mosquitoes that are either unfed (*M*_*uf.d*_), or blood fed (*M*_*f,d*_), and non-fatal attacks, which are represented by live mosquitoes that are blood fed (*M*_*f.l*_). It does, however exclude the unfed mosquitoes that remained alive (*M*_*uf,l*_), which in this case are considered to be the ones which did not attack the host. Equation 5 can therefore be broken down as follows:

(6) $$\begin{array}{l} \text{Δindoors}=1-\frac{\text{Muf},d+\text{Mf},d+\text{Mf},l}{\text{Mp}}\\ \;=\frac{\text{Muf},l}{\text{Mp}} \end{array}$$

Similarly, we have previously explained that in a single mosquito feeding cycle, attack related mortality can occur either before (*μ*_*pre*_) or after successful feeding (*μ*_*post*_) [11]. In practice both *μ*_*pre*_, and *μ*_*post*_ can be calculated directly from experimental hut data as fractions of the number of mosquitoes that attacked the host inside the huts.

(7) $$\text{μpre}=\frac{\text{Muf},d}{\text{Mp},\gamma }$$

(8) $$\text{μpost}=\frac{\text{Mf},d}{\text{Mp},\gamma }$$

where M_p,γ_ represents the total number of mosquitoes that entered the hut and did not divert from attacking and therefore either successfully fed or died in the attempt:

(9) $$\begin{array}{l} \text{Mp},y=\text{Mp}-\text{Muf},l\\ \;=\text{Muf},d+\text{Mf},d+\text{Mf},l \end{array}$$

The combined probability of attack related mortality is calculated as the proportion of all attacks that are fatal.

(10) $$\begin{array}{l} \mu =\text{μpre}+\text{μpost}=\frac{\text{Muf},d+\text{Mf},d}{\text{Mp},\gamma }\\ \;=\frac{\text{Mp},\text{dead}}{\text{Mp},\gamma } \end{array}$$

where *M*_*p,dead*_, refers to the total number of dead malaria mosquitoes caught inside the hut. In earlier versions of this model, these mortality probabilities (*μ*_*pre*_ and *μ*_*post*_) were combined and treated as a single event, assumed to occur prior to feeding [15,16,25]. This approach remains epidemiologically valid and relevant for most contemporary interventions, given that the post feeding mortality (*μ*_*post*_), which in practice is often measured as mortality within 24 hours, usually occurs within such a short time that those mosquitoes would not have possibly completed the gestation period, returned to a host seeking state or gone ahead to transmit disease to the next host anyway [26]. Moreover, the subdivision of attack-associated mortality into these two components is not necessary for estimating purely community-level protection against transmission, which unlike personal protection is a direct function of overall mortality probability *(μ)*[14]. This is to say, that while insecticide-related mosquito mortality occurring after the mosquito has fed on the protected host does not contribute to personal protection, it does contribute to community-level suppression of malaria transmission by reducing population mean mosquito survival.

Therefore to fulfil the current objectives, previous interpretations of the terms [15] are retained so that the probability of mosquitoes feeding upon an encountered host *(ϕ)* using a given protection measure *(p)* is expressed on the basis of both attack probability and the overall mortality probability:

(11) $$\varphi =\gamma \left(1-\mu \right)=M_{f,l}/M_{p}$$

The amendments above effectively render equations 10, 11 and 13 in our previous version of the model [11], unnecessary as the values needed to simulate effects of the interventions are no longer represented by additional probabilities of diversion (θ_Δ_) and death before feeding (θ_μ,pre_) caused by the deterrent and insecticidal properties of the nets respectively. Instead, the diversion (Δ) and mortality (μ) parameters are calculated directly from the experimental hut observations as described above.

As described previously [11,14], we fixed the true placebo baseline *(p = 0)* values for diversion *(Δ*_*0*_*)* and mortalities *(μ*_*0*_*)* at very low values (Δ_0_ = μ_0_ = 0.1) that are consistent with historical studies of houses and humans lacking any form of protection whatsoever [27]. Here, however, we explicitly consider protective effects of untreated nets [13,28], which are considerably greater than the baseline protection that results purely from individual defences of a person not using any protection at all [29], even though these nets are commonly used as controls in experimental hut studies [19]. Other than the highlighted changes, the rest of the equations remain exactly as described in the most recent version of this model [11].

### Input parameter values

The basic ecological parameter values used in this model version are similar to the most recent application [11]. The baseline diversion and baseline mortality probabilities for unprotected hosts were assumed to be 0.1 as in previous model applications [11,15], based on historical reports from true negative controls. As a representative epidemiological scenario, we simulated a closed community where residents own a small number of cattle and the malaria vector is *An. arabiensis*, which is an increasingly dominant vector species in Africa whose behavioural characteristics remain a significant challenge even after mass coverage with LLINs [10,13,30]. Recent studies in a rural Tanzanian village that was previously dominated by *An. gambiae s.s.* but now has mainly *An. arabiensis* as the malaria vector, have shown that over the years, the proportion of transmission that occurs indoors has changed from about 90% prior to the period of high LLINs coverage [31], to 0.79 in the years following high LLIN coverage [12,13]. The near complete elimination of *An gambiae s.s*, which was previously considered the most important vector, from these areas has been attributed to widespread use of insecticidal bed nets [12,13]. As such, we consider the proportion of exposure among unprotected persons that occurs at times when nets are normally in use (π_i_) to be 0.79. The other ecological difference relative to previous versions of the model was with regard to the ratio of human to cattle population, which we reduced to 10:1.4 as compared with the 1:1 ratio used previously [11], to reflect the latest cattle census data obtained by Health and Demographic Surveillance System at the Ifakara Health Institute, in the Kilombero valley (Additional file 1). All the simulations are also provided as supplementary online material (Additional file 1).

To represent the simulated interventions, the following specific changes were made on parameter values: first it was assumed that the total number of mosquitoes entering a house with no intervention at all is approximately equal to the total number entering huts with only untreated bed nets, as shown in equation 2 *(M*_*0*_ *≈ M*_*1*_*)*. Thus the *M*_*1*_ and other *M*_*P*_ values were obtained directly from experimental huts fitted with either untreated nets or other interventions. The malaria mosquitoes caught in the different experimental huts were classified as unfed and dead (*M*_*uf.d*_), fed and dead (*M*_*f.d*_), fed and alive (*M*_*f.l*_), or unfed and alive (*M*_*uf.l*_) and the values were inputted directly into model equations (Additional file 1). Since in most cases the feeding rates were so low that the estimated measures of central tendency would always be zero or near zero, we opted to use actual numbers of mosquitoes as recorded directly from the experimental huts, rather than the means or medians. To be consistent with globally agreed targets [1,32,33], the intervention coverage (i.e. proportion of people using the intervention) was set to 80%, equivalent to 800 of the 1000 people in this simulated community. Where both LLINs and IRS are used, the 80% intervention coverage refers to a situation where 80% of the people in the simulated community live in sprayed houses and the same people also sleep under nets, so that 20% of the population are considered unprotected in any given scenario. The main parameters and their respective values are described in Table 1.

**Table 1:** Main parameters and parameter values used in the evaluation

	**Description**	**Source of values**
Δ_outdoors_	The diversion that occurs outside the house when the mosquito is attempting house entry	Derived
Δ_indoors_	The diversion that occurs indoors when the mosquito has already entered the house to attack the human indoors	Derived
*M*	The total number of malaria vectors caught in a given human occupied hut	Implied
*M*_*0*_	The total number of malaria vectors caught in a given hut or set of huts having no protective treatment inside.	Actual numbers of female *Anopheles arabiensis* mosquitoes caught in unsprayed experimental huts fitted with untreated nets (Okumu *et al.,* unpublished). *M*_*0*_ values are considered equivalent to *M*_*1*_
*M*_*1*_	The total number of malaria vectors caught in a given hut or set of huts having untreated mosquito nets as the only form of protection inside. Subscripts *2….n* can be used to denote any other protective measures apart from untreated nets denoted by subscript ‘*1*’	
*M*_*p*_	The total number of malaria vectors caught in a given hut or set of huts having a protective treatment inside. Subscripts *2….n* can be used to denote any other protective measures apart from untreated nets denoted by subscript ‘*1*’ in *M*_*1*_	Actual number of female *An. arabiensis* caught in experimental huts that have different LLINs, IRS insecticides or various combinations of different LLINs and different IRS insecticides.
*M*_*p,y*_	Total number of malaria vectors that are considered to have entered the huts and attacked the humans inside the huts. They include female malaria vectors that are unfed and dead, blood fed and alive or blood fed and dead	
*M*_*u,.l*_	Total number of malaria vectors that are caught unfed and were still alive after 24 hours. Classifiable as non-attacking vectors	
*M*_*uf,d*_	Total number of malaria vectors that were caught unfed but died within 24 hours. Classifiable as fatal attacks	
*M*_*f,l*_	Total number of malaria vectors that were caught when already blood fed and remained alive after 24 hours. Classifiable as successful attacks	
*M*_*f,d*_	Total number of malaria vectors that were caught when already blood fed but died within 24 hours. Classifiable as fatal attacks	

The basic ecological parameter values used in this model version are similar to the most recent previous application [11]. However, all new or modified parameters are included here.

### Simulated interventions

The intervention data used here was obtained from an experimental hut study conducted in southern Tanzania (Okumu *et al.,* unpublished), where four net types (three LLINs and a non-insecticidal net) and three IRS insecticides of different classes (one organochloride, one >synthetic pyrethroid, and one organophosphate), were evaluated either singly or in combinations. The LLINs included Olyset® nets (manufactured by A-Z, Tanzania), PermaNet 2.0® nets (Vastergaard, Switzerland) and ICON Life® nets (supplied by Syngenta, Switzerland), which has similar specifications as the one marketed under the brand name, NetProtect® (Bestnet Europe Ltd, Denmark) [34]. Olyset® nets are polyethylene (150 denier), impregnated during manufacture with synthetic permethrin at 2% w/w (equivalent to 1000 mg of active ingredient/m^2^). PermaNet 2.0® is 100%-polyester (100 denier), coated with 55-62 mg of synthetic deltamethrin/m^2^, resulting in insecticide concentrations of approximately 0.14% w/w, depending on mesh size. Icon Life® is also polyethylene (118 denier), impregnated during manufacture with synthetic deltamethrin at 0.2% w/w (approximately 65-79 mg of active ingredient/m^2^ depending on mesh size).

The IRS chemicals included: 1) an organochloride, 75% pure DDT wettable powder (AVIMA, South Africa) sprayed at 2 g/m^2^ concentration of the active ingredient (a.i), 2) a synthetic pyrethroid, 10% capsule suspension of lambda-cyhalothrin brand named Icon 10 CS (Syngenta, Switzerland), sprayed at 0.03 g/m^2^ a.i, and 3) an organophosphate, 50% emulsified concentrate of pirimiphos-methyl also known as Actellic EC (Syngenta, Switzerland), sprayed at 2 g/m^2^ a.i. The IRS compounds and all the LLINs except Icon Life®, have been approved by WHO [35], and therefore represent a diversity of common insecticidal interventions currently applicable for vector control in Africa [20]. The data used here had been collected over a six month period after initial hut spraying (Okumu *et al.,* unpublished), which would translate to two applications per year, and are therefore comparable to plausible re-spraying rates for most IRS campaigns.

**Figure 2:**
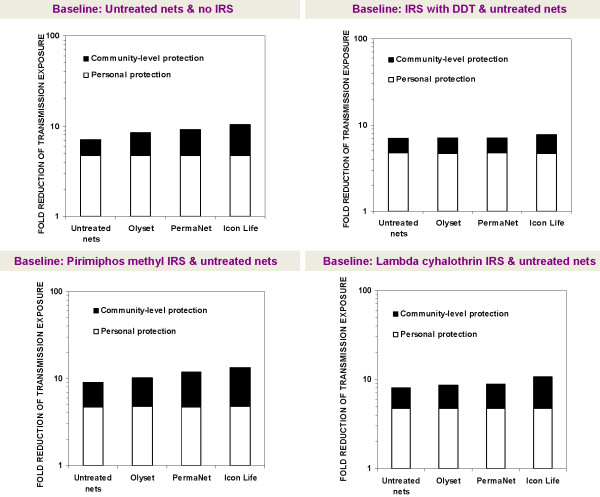
**Relative change in personal and community level protection, whenever LLINs are introduced into communities with pre-existing high coverage of IRS and untreated nets.** The labels on the x-axis refer to the additional complementary intervention in each scenario. The relative change can be interpreted as fold reduction of residual transmission, which is equivalent to the reciprocal of the relative residual EIR (entomological inoculation rate) for an average community member.

**Figure 3:**
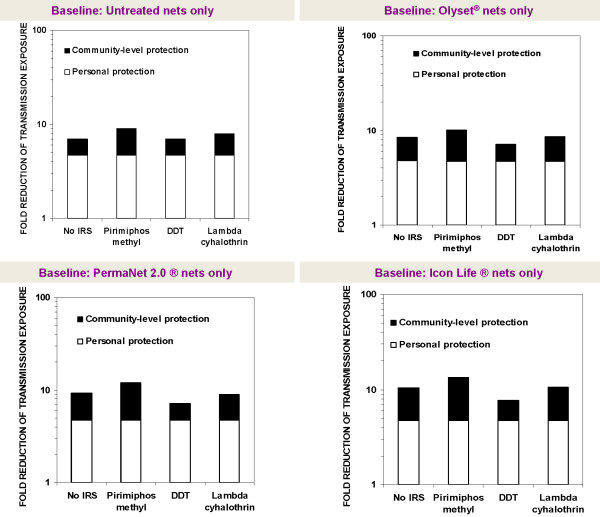
**Relative change in personal and community level protection, whenever IRS is introduced into communities with pre-existing high coverage of untreated nets or long lasting insecticide treated nets.** The labels on the x-axis refer to the additional complementary intervention in each scenario. The relative change can be interpreted as fold reduction of residual transmission, which is equivalent to the reciprocal of the relative residual EIR (entomological inoculation rate) for an average community member.

To examine whether combination of any of these LLINs with any of the IRS would lead to improved community-level epidemiological benefits relative to IRS alone or LLINs alone, we simulated two different situations: 1) where people were already using nets, so that IRS was considered the complementary intervention, and 2) where people are already using IRS with untreated nets, in which case the LLINs were considered the complementary intervention. For each complementary intervention, we calculated the relative improvement in malaria transmission control, in terms of the fold improvement in personal and communal protection that users obtain. For example, someone interested in the effects of adding Olyset® nets to communities already having DDT would compare transmission control achievable with DDT for IRS combined with Olyset® nets versus control achievable with just DDT used with untreated nets. Similarly, one may compare lambda cyhalothrin combined with PermaNet 2.0® versus PermaNet 2.0® nets used alone. The fold improvement in protection achievable by these interventions can also be interpreted as fold reduction of residual transmission, which is equivalent to the reciprocal of the relative residual EIR (entomological inoculation rate) as used in previous publications [11,14,15] and is calculated as the estimated mean EIR for communities with just the baseline intervention divided by the mean EIR for communities with respective LLIN/IRS combinations. These fold improvements in protection are presented graphically on logarithmic scales so that multiplicative effects of treating nets and combining them with IRS are represented additively in proportion to their incremental rather than absolute effects (Figures 2 and 3).

### Ethical approval

The interventions data used to parameterise this model was obtained from an experimental hut study where human participants volunteered to sleep inside huts as baits for adult mosquitoes. After full explanation of purpose and requirements of the studies, written informed consent was sought from each volunteer prior to the start of all experiments, and the volunteers received compensation for their time. While inside the experimental huts, the volunteers slept under intact bed nets as a basic protection against mosquito bites and were also provided with long sleeved, hooded jackets to provide additional protection from bites, whenever the volunteers stepped outside the nets to collect mosquitoes from interception exit traps attached to the huts. In addition, the volunteers were provided with access to diagnosis for malaria parasites using rapid diagnostic test kits, and treatment with the current first-line malaria drug (artemether-lumefantrine) in case they had malaria. Ethical approval for this work was granted by the Institutional Review Board of the Ifakara Health Institute (IHRDC/IRB/No.A019), the Tanzania National Institute of Medical Research (NIMR/HQ/R.8aNo1.W710) and the London School of Hygiene and Tropical Medicine (Ethics Clearance No. 5552).

## Results

This *in-silico* assessment showed that combining LLINs and IRS does not always result in improved community level malaria transmission control relative to the use of either method alone. Instead it was apparent that whereas introduction of LLINs into communities with pre-existing high coverage of IRS supplemented with untreated nets alone generally provide modest improvement in community level protection, adding IRS into communities with pre-existing high LLIN use is likely to be redundant or regressive except for modest improvements where the IRS compound is a highly mosquitocidal non-pyrethroid, as is predicted for LLINs plus pirimiphos methyl IRS relative to the LLINs alone. Figures 2 and 3 illustrate the specific fold enhancements of personal and community level protection where the pre-existing intervention is either LLINs used alone, or IRS supplemented with only untreated nets. The results also show that the overall impact of combining LLIN and IRS or untreated net and IRS is mainly due to the personal protection provided by the nets.

Where IRS is not applied but most people use intact untreated nets, replacing these untreated nets with Olyset®, PermaNet 2.0® or Icon Life® nets would variably improve community level protection against *An. arabiensis* mediated transmission, achieving predicted 3.7 fold, 4.4 fold and 5.7 fold enhancements compared with a 2.3 fold enhancement of protection achievable with untreated nets alone, when compared to situations with no protection at all (Figure 2). Approximately the same marginal to modest enhancements were predicted in situations where the pre-existing intervention consists of untreated nets plus IRS with either pirimiphos methyl or lambda cyhalothrin, but there was no predictable improvement in community level protection when any of the three LLINs are added into communities where most houses are already sprayed with DDT (Figure 2). Generally, the DDT based combinations also had the least impact upon *An. arabiensis* mediated transmission (Figures 2 and 3).

Relative to LLINs alone, all combinations of IRS and LLINs were predicted to offer little incremental protection except where pirimiphos methyl is used for IRS, but even in such cases, the predicted improvements were modest relative to what is achievable with LLINs alone. It was predicted that spraying houses with DDT would be redundant where most people already use intact untreated nets and would undermine protection achieved with LLINs alone when added as a supplementary intervention (Figure 3), presumably as a result of repelling mosquitoes away from houses [36] before the fatal contact can be achieved [11]. Spraying houses with lambda cyhalothrin would be redundant where most people already use any of the three LLINs, but the same IRS would marginally improve communal protection when added into communities with high coverage of untreated nets (Figure 3). Pirimiphos methyl was the only IRS compound predicted to achieve at least some modest enhancements of communal protection relative to nets alone. The insecticide would achieve a 4.3 fold improvement in community protection compared to 2.2 fold improvement achieved with untreated nets, 5.4 fold improvement when used together with Olyset® nets compared to 3.6 fold reduction achievable with Olyset® nets alone, and an 8.6 fold improvement when combined with Icon Life® nets compared to a 5.7 fold improvement achievable when the Icon Life® nets are used alone (Figure 3).

## Discussion

Overall, the surprising trend throughout all the primary experimental hut observations (Okumu *et al.,* unpublished) and these simulations (Figures 2 and 3) is that chemical insecticides, whether applied as active ingredients for IRS or LLINs, add minimal additional personal protection and, at best, only modest additional community-level protection relative to that achieved by the purely physical barrier of intact but untreated nets. The direct implication of these simulations is that improving the robustness of netting materials may be more important to maximizing the limited protection nets can provide against malaria transmitted by *An. arabiensis*, rather than optimizing insecticide formulations for treating them or for supplementing them with IRS. However, the greatest limitation of any entomological survey or experiment in a setting such as this where control with IRS or LLINs have been successful is simply that the most important target species which have been successfully suppressed are absent or rare [12,13,37,38]. The vector species most dramatically affected by scale up of LLINs or IRS are the most anthropophagic, human-dependent species which are correspondingly the most potent vectors [26,39-42]. Correspondingly, those that persist are usually those that were always least amenable to control with LLINs or IRS in the first place because of pre-existing behavioural resistance traits such as zoophagy and exophagy. Such experimental hut studies, and derived simulations, of IRS and LLIN impact upon residual vector populations and may therefore be most appropriately interpreted in terms of defining the limits of achievable impact upon transmission by such resilient species as *An. arabiensis*.

The empirical and theoretical studies described here clearly illustrate why behaviourally resilient *An. arabiensis*[13,37,38,43,44] increasingly dominate residual vector systems across east Africa, and suggest that transmission by this species may not be equally as amenable to control by LLINs and IRS as the endophillic and endophagic sibling species, *An. gambiae s.s.*[12,13,38,44]. Therefore, while direct interpretation of these results at face value might appear discouraging in relation to the value of LLINs and IRS, the simple fact that *An. arabiensis* was the only vector these relate to indicates they have had a massive impact upon the more potent sibling species *An. gambiae* in this setting. The dramatically modified overall composition of residual malaria vector populations across east Africa points to enormous and consistent success of LLINs in particular against the *An. gambiae s.s.* which historically dominated transmission in most of the region, because they predominantly rest and feed indoors. While the disappointing results presented here relate only to *An. arabiensis*, we expect that the minor incremental improvements observed for this species, such as those that are seen when lambda cyhalothrin is added to houses with LLINs, will be manifested as far greater differences for *An. gambiae s.s.* or *An. funestus s.s.*.

It might therefore be dangerous to over-extend interpretation of these results beyond situations dominated by *An. arabiensis* because any de-prioritization of LLINs and IRS could possibly allow both *An. gambiae* and *An. funestus* populations to rebound to historical levels. The paradox of absent or sparse mosquito populations remaining the highest target priority however presents a methodological new conundrum, which needs to be urgently considered. That is to say, how do we monitor, optimize, manage and sustain vector control interventions that have been so successful that their primary target species are now too rare to study in the wild? While it is possible to wait until such priority target populations recover to historical norms following the emergence of physiological or behavioural resistance, such a defeatist strategy would inevitably result in long periods of intervention failure and potentially a situation of overwhelming malaria resurgence. Equivalent experimental hut assays inside large cage systems with captive, self-propagating populations [45] may represent one of the only strategies available for testing new intervention options against these species [26,46].

Whereas these simulations predict very limited value of adding IRS onto LLINs, there could still be a few scenarios under which combinations of the two interventions would significantly enhance the community level transmission control relative to IRS alone or untreated nets alone, even where the dominant vector is *An. arabiensis*. For example, from a practical point of view, one would expect improved benefits from such combinations in areas where the nets do not remain intact for long, where the nets are not used consistently, and also where there is the rapid decay of IRS compounds coupled with inconsistent re-spraying programs [47]. In such cases, LLINs could extend the temporal protective coverage even after the IRS insecticides have decayed, while IRS on the other hand could confer additional protection to people using torn nets or people not consistently sleeping under their nets, especially if mosquitoes successfully feed upon the net users and then rest on the sprayed house surfaces [3], and if the IRS chemical is adequately toxic so that it can kill mosquitoes even on very short contact with treated surfaces. Even though these possibilities are not captured in the current simulations, it is reasonable to subject them to alternative viewpoints and expert discussion and to recognize that since our parameter estimates came from an experimental hut trial this model may slightly underestimate the real value of LLIN-IRS combinations in practical situations.

Nevertheless, because any appreciable enhancement of communal protection is only likely with non-pyrethroid IRS insecticides, the decision to implement LLIN-IRS combinations must also be more carefully evaluated on the basis of available resources. Selecting the best LLIN types and LLIN properties against *An. arabiensis* can therefore be considered another useful outcome of this work, which could enable optimal use of resources for net distribution programmes. In the same regard, supplementing nets with IRS offers relatively modest incremental benefits (Figure 3), but given the substantial costs of implementing adulticide-based vector control programmes [48], it is unlikely that those marginal benefits would be greater than the marginal costs of adding the complementary intervention. Where possible, LLINs coverage should therefore be expanded and consistent use ensured through community education and regular net replacement, before attempting to also provide IRS. It is also worth noting that with regard to adding IRS onto LLINs, the only IRS insecticides, among the evaluated candidates, that would provide at least modest enhancement of communal protection, i.e. pirimiphos methyl, is also the one that would be most expensive in terms of unit cost and dosage of application [49]. On the other hand, the use of pyrethroids for IRS in addition to LLINs, all of which are also pyrethroid-based should be discouraged as this would possibly accelerate the rise and spread of physiological insecticide resistance among malaria vector populations [50]. Instead, addition of IRS with organophosphate (pirimiphos methyl) or carbamates, may actually be preferable as an insecticide resistance management strategy even in the epidemiological settings simulated here [50].

This report includes a revised model formulation that offers several advantages and opportunities to vector control researchers and practitioners. Perhaps the most important advantage is the sub-division of mosquito diversionary processes into indoor and outdoor compartments, so that untreated mosquito nets can be used as a negative pseudo-control with which to estimate spatial repellency of treated nets, and can also be evaluated themselves in terms of the protective effects they exert against mosquitoes that enter houses. The model formulation presented here also has particular utility, because it is specifically designed to be directly parameterized from standardized experimental hut assay result records (WHO 2006). However, it should be noted that this is only possible if such experimental hut studies report all the necessary input parameters, with the M_uf,l_, M_uf,d_, M_f,l_ and M_f,d_ quantities all separately and explicitly provided. Separate values for M_uf,d_ and M_f,d_ are rarely reported yet they are particularly important because the sub-division of mortality processes is particularly essential for modelling of insecticidal interventions of varying modes of action. This could be particularly useful and important when comparing interventions that are known to exhibit fast acting toxicity, i.e. those that can kill mosquitoes immediately on attack, versus those that are known to be slow acting [11] i.e. those that exhibit delayed toxicity to mosquitoes, e.g. fungal bio-agents [51] or insecticides such as chlorfenapyr [6,52]. Moreover, where blood fed mosquitoes remain indoors and rest on walls it is likely that IRS with commonly used existing insecticide formulations would elicit mostly post-feeding mortality.

One possible limitation of these simulations is that they are based on the assumption that the nets and the IRS are used in the best way possible. For instance, we have used the data from our experimental hut studies where volunteers always used the nets consistently, and also where all the nets were new and not torn. As a result, we observed that there were a very small proportion of fed mosquitoes in the huts (Okumu *et al*., unpublished). In practice, nets may often get torn, thereby increasing the likelihood that mosquitoes obtain blood meals from the human volunteers in the experimental huts. This would in effect lower the protective efficacy of the nets. Therefore, in order to actually achieve this simulated potential, all LLINs would need to be maintained in an intact insecticidal state, possibly by replacing the nets every one or two years.

Although it is apparent that the physical barrier effect of intact nets could already confer significant impact on *An. arabiensis* vector populations, even if the nets have no insecticides, it is important to realize that any of the personal protection achievable with intact untreated nets or intact LLINs is only relevant as far as indoor transmission is concerned. This is to say, even though intact nets could provide up to 99% protection from bites (Okumu *et al.* unpublished), in reality they would provide only 78%, given the reduced proportions of indoor malaria transmission that occurs in communities dominated by *An. arabiensis* (in this case, π_i_ = 0.79) [12]. Moreover, this level of protection is likely to continue falling, as more and more malaria transmission occurs outdoors. This also means that whereas the best intradomicilliary options we have today against vectors like *An. arabiensis* remain intact LLINs (acting mainly as physical barriers and only modestly as mosquitocidal interventions), there is still need for complementary interventions [30,53] to provide coverage against the other proportion of the residual malaria transmission that occurs outside the spectrum of net efficacy, e.g. early in the evening or outdoors.

## Conclusion

Where malaria transmission is mediated primarily by *An. arabiensis*, introduction of LLINs into communities with pre-existing high coverage of IRS plus untreated nets generally contribute modest improvements in community level protection, but introduction of IRS into communities with pre-existing high LLIN use is in most cases redundant except where the IRS compound is a highly mosquitocidal non-pyrethroid like pirimiphos methyl, which surprisingly also offers only modest improvements relative to the LLINs alone. The overall impact of combining LLIN and IRS or untreated net and IRS is, however, mainly due to the personal protection provided by the nets, rather than insecticidal efficacy. While IRS with the organophosphate, pirimiphos methyl, or other non-pyrethroid insecticides with similar properties, may offer the best option for enhancing the impact of intact pyrethroid LLINs upon *An. arabiensis*, these incremental benefits are modest and therefore a stronger rationale for such an expensive combination would be to address deficits in net durability and usage, to prevent rebound of indoor feeding and indoor resting vectors that could mediate high malaria transmission, as well as to ensure pre-emptive insecticide resistance management. Improving the robustness of netting materials may be more important to maximizing the limited protection nets can provide against malaria transmitted by *An. arabiensis*, rather than optimizing insecticide formulations for treating them or for supplementing them with IRS. These results clearly outline the fundamental limits of IRS and LLINs for controlling *An. arabiensis* but these conclusions cannot be applied to more anthropophagic, potent, behaviourally vulnerable and historically important vector species such as *An. gambiae* and *An. funestus*. The recent decline of these more important target species confirms that they remain vulnerable to LLINs and IRS, which should therefore be sustained and optimized in the long term to prevent possible rebound.

## Competing interest

While this study was independently funded primarily through the U.S. President’s Malaria Initiative via United States Agency for International Development (USAID), with other support awarded to SJM and FOO as stated above, two of the authors have also previously received support for other research projects from manufactures of insecticidal public health products: Vestergaard Frandsen SA (GFK) and Syngenta (SJM).

## Authors’ contributions

FOO and GFK wrote the mathematical model. FOO and SJM extracted the parameter values from the experimental hut trials. FOO, SK and GFK implemented the simulations. FOO drafted the original manuscript. All authors participated in the interpretation of the result and approved the final version before submission.

## Supplementary Material

Additional file 1 An excel spreadsheet showing actual model simulations, input parameters and outputs. Click here for file
